# Dorsal Pancreatic Agenesis Associated With New‐Onset Diabetes Mellitus and Acute Pancreatitis in an 18‐Year‐Old Man: A Case Report

**DOI:** 10.1002/ccr3.73606

**Published:** 2026-09-28

**Authors:** Mugheesa Rab, Muhammad Hassaan Javaid, Rabia Narmeen Numani, Fozia Khanam, Muddassir Khalid, Fred Segawa

**Affiliations:** ^1^ Department of Medicine Rawalpindi Medical University Rawalpindi Pakistan; ^2^ Department of Medicine Shifa College of Medicine Islamabad Pakistan; ^3^ Department of Radiology Nishtar Medical University Multan Pakistan; ^4^ Department of Medicine Nishtar Medical University Multan Pakistan; ^5^ Department of Medicine Makerere University College of Health Sciences Kampala Uganda

**Keywords:** acute pancreatitis, dorsal pancreatic agenesis, MRCP, new‐onset diabetes mellitus, pancreatic anomaly, pancreatogenic diabetes

## Abstract

Dorsal pancreatic agenesis (DPA) is a rare congenital disorder resulting from failure of development of the dorsal pancreatic bud. Complete DPA implies absence of the pancreatic neck, body, and tail and can be associated with both diabetes mellitus and pancreatitis. The patient was an 18‐year‐old man admitted with a history of epigastric pain, nausea, vomiting, polyuria, polydipsia, and unintentional weight loss of 4 kg over 8 weeks. The lab investigations included elevated serum glucose (412 mg/dL), urine ketones 3+, venous pH 7.31, bicarbonate 17.2 mEq/L, anion gap 14.8 mEq/L, lipase 486 U/L, amylase 312 U/L, consistent with hyperglycemic ketosis and metabolic acidosis, treated as mild DKA. The contrast‐enhanced computed tomography (CT) study was read in real time by an abdominal radiologist and demonstrated the absence of the pancreatic neck, body, and tail, increased size of the head of the pancreas, a positive dependent‐stomach sign, and mild pancreatic inflammation without necrosis and fluid collections. MRCP done 8 weeks later confirmed a normal ventral pancreatic duct and absence of dorsal pancreatic duct, consistent with the diagnosis of complete DPA. Fasting C‐peptide 12 weeks after presentation was found to be extremely low (0.31 ng/mL) and antibodies to GAD65, IA‐2A, and ZnT8 negative. These findings decreased the likelihood of classic autoimmune type 1 diabetes, suggesting involvement of the underlying pancreatic pathology and the presence of severe insulin deficiency. However, negative antibody type 1 diabetes cannot be ruled out. DPA should be suspected in young patients with unexplained pancreatitis and insulin‐deficient diabetes. Imaging is essential, and MRCP provides the most valuable ductal information. Classification of diabetes should not depend solely on age, ketosis, or insulin requirement when a major congenital abnormality of the pancreas is present.

## Key Clinical Message

Dorsal pancreatic agenesis (DPA) must be considered in young patients presenting with pancreatitis and diabetes without insulin secretion. The former can be diagnosed based on morphologic appearance by CT, whereas MRCP confirms the ductal system abnormalities. Low C‐peptide and negative islet cell antibodies contribute to the diagnosis of diabetes.

## 1 Introduction

Dorsal pancreatic agenesis (DPA) is a rare congenital anomaly of the pancreas caused by failure of development of the dorsal pancreatic bud. The dorsal bud usually forms most of the body and tail of the pancreas, while the ventral bud mainly forms the head of the organ and its uncinate process [1, 2, 3]. Consequently, total absence of the dorsal bud causes absence of the distal portion of the pancreas, while the head of the pancreas persists. It may either be a silent disease or one that is accompanied by abdominal pain, pancreatitis, diabetes mellitus, or exocrine pancreatic insufficiency. The development of diabetes in these patients may be due to the lack of a substantial portion of endocrine tissue [1, 2].

The diagnosis depends upon imaging. CT scan shows lack of the body and tail of the pancreas, enlarged head of the pancreas, as well as the presence of the dependent stomach or intestine; usually the enlargement is thought to be due to compensatory hypertrophy; however, inflammation‐related edema might also have influence here. MRCP gives us more information regarding the pancreatic duct system and allows us to distinguish between congenital absence of the pancreas and acquired atrophy of the pancreas.

In our case, an 18‐year‐old male patient with DPA (according to CT morphology and MRCP) who had pancreatitis and developed insulin‐dependent diabetes with hyperglycemia and ketosis and metabolic acidosis.

## 2 Case History

An 18‐year‐old male patient with no known past medical history of diabetes, pancreatic disease, abdominal trauma, or surgery had an acute onset of epigastric pain associated with nausea and vomiting. The patient had a history of intermittent mild abdominal pain after eating for about 2 months. In the last 8 weeks, he lost about 4 kg and also had polyuria and polydipsia. The clinical timeline of the case is given in Table 1.

**TABLE 1:** Clinical timeline.

Time	Clinical event
−8 weeks	Intermittent postprandial abdominal discomfort and approximately 4‐kg weight loss began
−8 to 0 weeks	Polyuria and polydipsia developed
Day 0	Acute epigastric pain, nausea, and vomiting
Day 0	Glucose 412 mg/dL, urinary ketones 3+, bicarbonate 17.2 mEq/L, lipase 486 U/L, amylase 312 U/L
Early hospitalization	Intravenous fluids, insulin, analgesia, electrolyte monitoring, and supportive treatment
During hospitalization	Contrast‐enhanced CT demonstrated absence of pancreatic neck, body, and tail with enlarged pancreatic head and peripancreatic inflammation
Discharge	Basal‐bolus insulin therapy initiated
Week 8	MRCP demonstrated preserved ventral duct and absent dorsal pancreatic duct
Week 12	HbA1c 7.1%, fasting C‐peptide 0.31 ng/mL; GAD65, IA‐2A, and ZnT8 antibodies negative
Week 12	Fecal elastase 210 μg/g; no steatorrhea; pancreatic enzyme replacement not initiated

The patient denied any alcohol, tobacco, or illicit drug use, recent medications, history of abdominal trauma, or prior episodes of pancreatitis. There was no significant family history of diabetes, chronic pancreatitis, pancreatic carcinoma, or other pancreatic diseases. His height was 172 cm, weight 58 kg, and BMI 19.6 kg/m^2^.

Clinical findings included stable vital signs and epigastric and right‐upper‐quadrant tenderness without guarding or rebound tenderness.

### 2.1 Differential Diagnosis

The differential diagnosis for apparent absence of the pancreatic body and tail includes pancreatic lipomatosis, chronic pancreatitis with distal atrophy, prior pancreatitis‐related necrosis or trauma, distal duct obstruction, pancreatic neoplasm, and congenital DPA.

Pancreatic lipomatosis represents a condition in which pancreatic tissue is replaced by fat.

Chronic pancreatitis can cause atrophy and ductal changes; however, there was no history of chronic inflammation.

The absence of any prior pancreatic injury or surgery excluded prior trauma or necrotizing pancreatitis.

A pancreatic tumor was considered unlikely given the patient's young age and the absence of a discrete mass or obstructive lesion.

Combined, the lack of the pancreatic neck, body, and tail; enlargement of the head of pancreas; dependent‐stomach sign; and lack of dorsal pancreatic duct on MRCP were the most compelling evidence for congenital DPA.

### 2.2 Investigations

Initial laboratory investigation revealed significant hyperglycemia with ketonuria and mild metabolic acidosis. Admission glucose was 412 mg/dL, urine ketones 3+, venous pH 7.31, bicarbonate 17.2 mEq/L, and anion gap 14.8 mEq/L. These findings are consistent with hyperglycemia ketosis with mild acidosis and have been treated clinically as mild DKA. Serum β‐hydroxybutyrate levels were not measured, and biochemical parameters reflecting biochemical recovery from the ketosis were not obtained and are recognized to be limitations. Initial laboratory investigations are given in Table 2.

**TABLE 2:** Principal admission laboratory findings.

Parameter	Patient value	Reference range
Glucose	412 mg/dL	70–140
Venous pH	7.31	7.32–7.42
Bicarbonate	17.2 mEq/L	22–29
Anion gap	14.8 mEq/L	8–12
Urinary ketones	3+	Negative
Lipase	486 U/L	13–60
Amylase	312 U/L	28–100
Triglycerides	184 mg/dL	< 150
Calcium	9.1 mg/dL	8.5–10.2
Sodium	134 mEq/L	135–145
Potassium	4.3 mEq/L	3.5–5.0
Chloride	102 mEq/L	96–106
Creatinine	0.89 mg/dL	0.70–1.30
Total bilirubin	0.7 mg/dL	0.3–1.2
Alkaline phosphatase	88 U/L	44–147

Serum lipase was 486 U/L (upper reference limit 60 U/L) and serum amylase was 312 U/L (upper reference limit 100 U/L), each exceeding the upper reference limit more than threefold. Together with typical epigastric pain and CT evidence of peripancreatic inflammation, this fulfilled two of the standard diagnostic criteria for acute pancreatitis [4].

The etiological work‐up showed triglycerides to be 184 mg/dL and calcium to be 9.1 mg/dL. The serum bilirubin, alkaline phosphatase, and aminotransferases did not indicate any bile duct obstruction. Gallstones and biliary dilatation were not found on CT scan. No history of alcohol consumption, trauma, or exposure to drugs was noted.

On ultrasonography, a large hypoechoic pancreatic head measuring approximately 38 mm was seen. The pancreatic body and tail were initially described as normal; however, on detailed retrospective review, it was determined that this reflected inadequate visualization of these structures rather than confident documentation of their normalcy. The apparent ultrasonographic–CT discrepancy is therefore attributable to inadequate ultrasonographic visualization of the pancreas rather than a true structural discrepancy.

Contrast‐enhanced 64‐slice CT was performed following intravenous administration of 100 mL of iohexol (350 mg iodine/mL), at a flow rate of 3.0 mL/s, and portal‐venous phase imaging was done at 65 s after the administration of contrast, with a reconstruction interval of 1.25 mm. The entire contrast enhanced examination was evaluated by an experienced abdominal radiologist in real time and the finding was absence of the pancreatic neck, body, and tail distal to the splenic vein; enlargement of the head of the pancreas (anteroposterior diameter 4.1 cm) with normal parenchymal enhancement; minimal peripancreatic fat stranding without any signs of necrosis, pseudocyst or organized fluid collection; and positive dependent stomach sign. There was no dilatation of the bile ducts or presence of any pancreatic mass.

In this particular case, the CT study was contrast‐enhanced and this information was included in the radiology report at the time of admission. Only the screenshots that were labeled as “plain scan” were kept in the institutional database and are reproduced in the present manuscript. These screenshots do not contain the information regarding the contrast phase and are not enough for independent evaluation of the pancreatic enhancement and the patency of the blood vessels. Archival retrieval of the contrast‐enhanced DICOM series was not possible. Accordingly, Figures 1 and 2 are shown here as representative archived images, whose legends have been modified to ensure that there is no claim made about contrast phase or patency (including splenic vascular patency). The conclusion about DPA based on this case report is based on the interpretation of the contrast‐enhanced imaging done by the radiologist along with MRCP, and not on the archived image alone.

**FIGURE 1:**
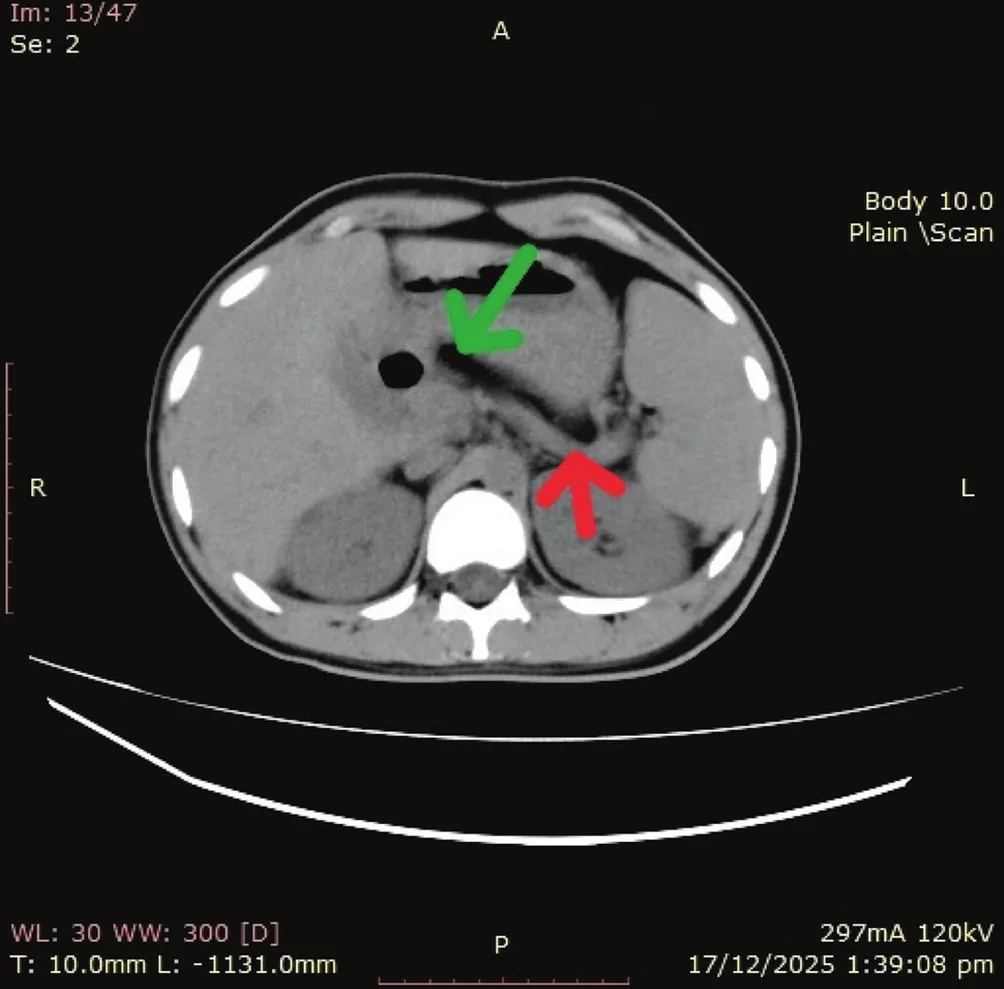
Contrast‐enhanced CT abdomen showing absence of the pancreatic body and tail (green arrows) with unobstructed splenic vessels (red arrow), consistent with dorsal pancreatic agenesis. This representative image was retrieved from the institutional archive labeled “Plain Scan”; however, the acquisition phase could not be independently verified, and no definitive statement regarding contrast enhancement or vascular (including splenic vessel) patency can be made.

**FIGURE 2:**
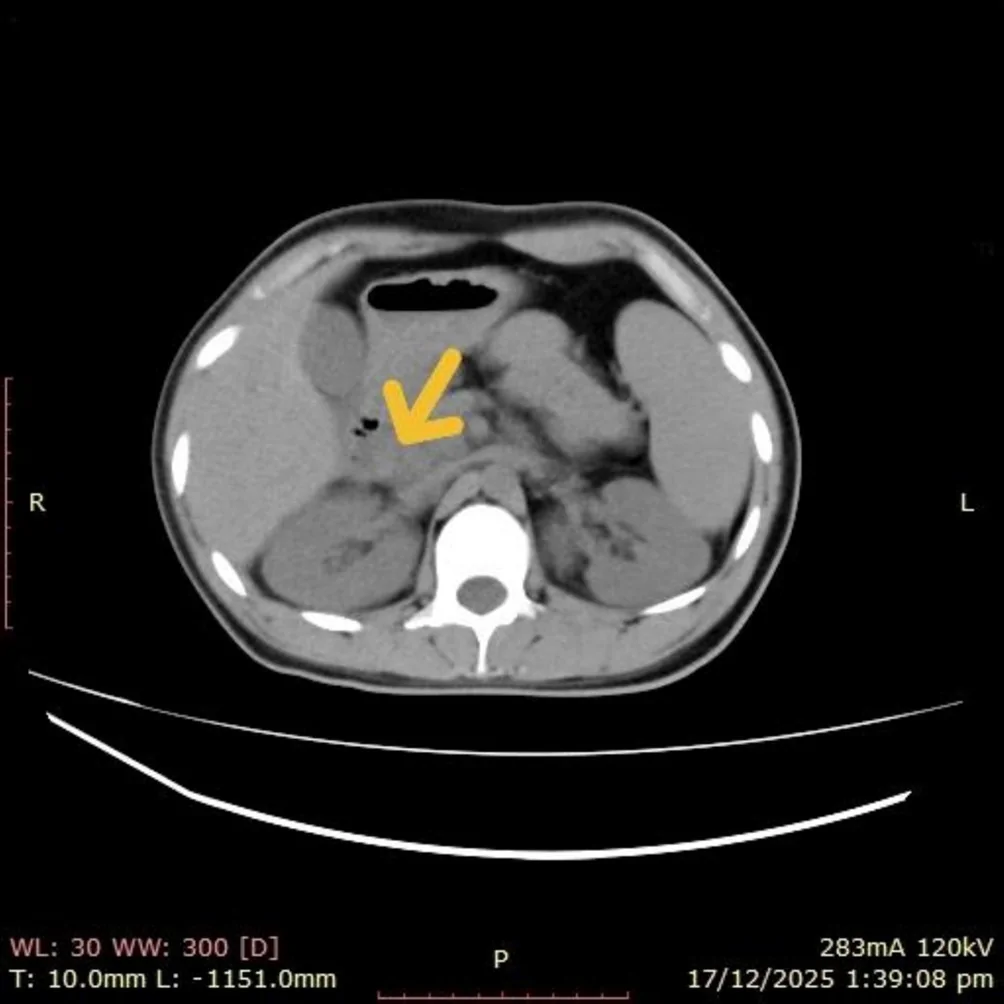
Contrast‐enhanced CT abdomen demonstrating hypertrophy of the pancreatic head (yellow arrow). This representative image was retrieved from the institutional archive labeled “Plain Scan”; however, the acquisition phase could not be independently verified, and no definitive statement regarding contrast enhancement or vascular patency can be made.

MRCP performed 8 weeks after discharge revealed preservation of the ventral pancreatic duct (duct of Wirsung) and complete absence of the dorsal pancreatic duct (duct of Santorini), as well as CT imaging evidence of absence of the pancreatic neck, body, and tail. An MRCP image (thick slab or maximum intensity projection) could not be found among the archived documents to be included in the report; this is considered a limitation, and the document should be included if it can be found later on. Diabetes diagnosis changed once the clinical stabilization of the patient was achieved. The level of C‐peptide measured after 12 weeks was 0.31 ng/mL, thus confirming the low level of insulin production; however, the value of fasting glucose and the reference range for this test were not recorded, which is considered a limitation. GAD65, IA‐2A, and ZnT8 autoantibodies were negative. These results suggest that classic type 1 diabetes due to autoimmune reaction is unlikely; nevertheless, it cannot be completely ruled out. Taking into account the lack of clinically relevant exocrine pancreatic insufficiency (see below), the terms “pancreatogenic diabetes” and “type 3c diabetes” are used cautiously in this report, preferring the term diabetes with DPA and severe insulin deficiency.

Fecal elastase‐1 was 210 μg/g. The patient did not have steatorrhea or malabsorption.

### 2.3 Management

The conservative management included IV fluid resuscitation, use of insulin, analgesics, electrolyte measurement, and dietary restrictions along with gradual introduction of oral feeds, resulting in alleviation of the abdominal complaints. As the potassium level was 4.3 mEq/L at the time of admission, therefore, there was no need for potassium replenishment before initiating insulin therapy. The hyperglycemic and ketotic state improved with time during the stay in the hospital, and the patient was eventually discharged on a basal bolus insulin regimen (glargine insulin 10 units nocturnally and lispro insulin 4 units before major meals). The surgical intervention was not needed for pancreatitis and DPA. The pancreatic enzymes were not prescribed because the stool elastase level was 210 μg/g and there were no clinical manifestations of exocrine insufficiency.

### 2.4 Follow‐Up and Outcomes

By week 12, blood glucose while fasting was 110–140 mg/dL and 140–180 mg/dL postprandially. HbA1c decreased from 9.73% to 7.1%. He continued to use insulin and perform his own glucose measurements without incidents of hypoglycemia.

There was no recurrence of episodes of abdominal pain and vomiting as well as reoccurrence of pancreatitis. C‐peptide level in fasting state would still remain low at 0.31 ng/mL and GAD65, IA‐2A, and ZnT8 antibodies would be negative. Exocrine insufficiency was not evident (fecal elastase 210 μg/g). DPA was confirmed with MRCP at 8 weeks.

## 3 Discussion

In this scenario, both acute pancreatitis and newly diagnosed DM have been documented. Diabetes in DPA makes biological sense, in that reduction of pancreatic body and tail can decrease the number of beta cells. Absence of GAD65, IA‐2A, and ZnT8 antibodies along with C‐peptide 0.31 ng/mL decreased the probability of classic autoimmune type 1 DM, but type 1 DM negative for antibodies can be ruled out solely on this ground. Combined with the presence of a structural abnormality of the pancreas, the phenotype of diabetes in this patient is more indicative of a structural pancreatic insufficiency compared to the autoimmune process. But the assignment of this patient to type 3c diabetes has to be made cautiously because, in addition to the presence of a structural pancreatic abnormality, type 3c diabetes also requires a lack of any signs of autoimmunity and presence of exocrine insufficiency [5]. Since in this particular patient, exocrine insufficiency has not been established (fecal elastase 210 μg/g, no steatorrhea), classification of this particular patient as type 3c is not justified. Comparisons with published cases are given in Table 3.

**TABLE 3:** Comparison with selected published DPA cases.

Study	Patient/presentation	Diabetes	Pancreatitis/DKA	Imaging confirmation
Cienfuegos et al. [1]	Systematic review of 53 reported patients	Frequently reported	Pancreatitis reported in subset	Variable
Lalchandani et al. [6]	Adult with abdominal pain and diabetes	Present	No acute pancreatitis	CT/MRCP
Yang et al. [7]	30‐year‐old man	Present	DKA	CT/MRCP
Bhattarai et al. [8]	Adult patient	Insulin‐dependent	Variable	Imaging‐based
Present case	18‐year‐old man	Severe insulin deficiency; antibody‐negative	Acute pancreatitis with hyperglycemic ketosis/metabolic acidosis	CT morphology + MRCP ductal confirmation

The diagnosis of acute pancreatitis was supported by abdominal pain, pancreatic enzyme elevation more than threefold above the upper reference limit, and CT findings. Absence of alcohol use, hypertriglyceridemia, hypercalcemia, biliary obstruction, trauma, or causative medications made other etiologies of pancreatitis less likely [4].

Diagnostic radiology in the current case had certain peculiarities. DPA may mimic pancreatic lipomatosis, chronic pancreatitis, distal atrophy of the pancreas, or past pancreatic necrosis or injury. Features on CT such as the absence of the body and tail of the pancreas, pancreatic head hypertrophy, and dependent stomach/dependent intestine sign may serve for the diagnosis of DPA [3]. In the current patient, the dependent stomach sign helped make structural diagnostics. Because the CT was done during an episode of acute pancreatitis, the enlarged pancreatic head could be due to inflammatory edema rather than proven compensatory hypertrophy. In the absence of a previous or interval imaging showing that there is persistence of an enlarged head after the inflammation of pancreatitis subsides, “enlarged pancreatic head” is a better term than “compensatory hypertrophy”.

In particular, the first ultrasound was not able to reliably demonstrate the existence of the body and tail of the pancreas. The difference between CT and US was due to poor ultrasonographic imaging of the body and tail of the pancreas. MRCP demonstrated the complete absence of the dorsal pancreatic duct, which is normal. DPA may occur as part of developmental anomalies and genetic syndromes. Mutations in GATA6 are a recognized cause of human pancreatic agenesis, and recessive regulatory mutations in PTF1A have been described in isolated pancreatic agenesis [9, 10]. Genetic testing was not performed in this case, so no molecular mechanism could be attributed.

What makes this case significant is that there have been documented cases of DPA, DPA associated with diabetes, pancreatitis, and even diabetic ketoacidosis. This case is unique in the fact that complete DPA was found in conjunction with acute pancreatitis and severe insulin deficiency without the presence of antibodies in an 18‐year‐old individual. The significance of this case can also be attributed to the need for reconsidering the presumptive diagnosis of type 1 diabetes on the basis of pancreatic imaging, antibody studies, and C‐peptide findings.

## 4 Limitations

There are limitations in this case study. β‐hydroxybutyrate and other biochemical parameters used to assess recovery from ketoacidosis were not provided, and the information about administration of acute insulin and fluid treatment was unavailable. The paired measurement of the glucose concentration and reference range for 12‐week C‐peptide test was not available. No other than “plain scan”‐labeled CT pictures were available, which made independent analysis of contrast enhancement impossible. Genetic testing was not performed and follow‐up was limited to 12 weeks.

## 5 Conclusion

Complete DPA is an infrequent congenital pancreas abnormality that can be associated with pancreatitis and profound deficiency of insulin. The CT image shows the typical pancreas anatomy, while MRCP gives the ductal evidence. For patients with a severe defect in pancreas anatomy, the diagnosis of diabetes needs to consider imaging, C‐peptide level, and antibody evidence instead of the other parameters.

## Author Contributions

**Mugheesa Rab:** methodology, software, data curation. **Muhammad Hassaan Javaid:** writing – original draft. **Rabia Narmeen Numani:** conceptualization, writing – review and editing. **Fozia Khanam:** conceptualization, data curation, investigation, visualization, validation. **Muddassir Khalid:** writing – review and editing, project administration, supervision. **Fred Segawa:** resources.

## Funding

The authors have nothing to report.

## Ethics Statement

The authors have nothing to report.

## Consent

Written informed consent was obtained from the patient for publication of this case report and accompanying images. A copy of the written consent is available for review by the Editor‐in‐Chief of this journal on request.

## Conflicts of Interest

The authors declare no conflicts of interest.

## Data Availability

The data that support the findings of this study are available on request from the corresponding author. The data are not publicly available due to privacy or ethical restrictions.
